# “Analysis of readmissions to the emergency department among patients presenting with abdominal pain”

**DOI:** 10.1186/s12873-020-00334-x

**Published:** 2020-05-12

**Authors:** Artur Kacprzyk, Tomasz Stefura, Katarzyna Chłopaś, Kaja Trzeciak, Aleksandra Załustowicz, Mateusz Rubinkiewicz, Michał Pędziwiatr, Kazimierz Rembiasz, Piotr Major

**Affiliations:** 1 Jagiellonian University Medical College, 31-008 Cracow, Poland; 2 2nd Department of General Surgery, Jagiellonian University Medical College, Kopernika 21 St, 31-501 Cracow, Poland; 3 Centre for Research, Training and Innovation in Surgery (CERTAIN Surgery), 31-501 Cracow, Poland

**Keywords:** Abdominal pain, Emergency department, Readmission

## Abstract

**Background:**

Abdominal pain is one of the most common complaints among patients admitted to the Emergency Department (ED). Diagnosis and management of abdominal pain may be a challenge and there are patients who require admission to the ED more than once in a short period of time. Our purpose was to assess the incidence of readmissions among patients treated in the ED due to abdominal pain and to investigate the impact of readmission on the further course of treatment.

**Methods:**

We conducted a prospective observational study, which included patients admitted to the ED in one academic, teaching hospital presenting with non-traumatic abdominal pain in a three-month period. Analyzed factors included demographic data, details related to first and subsequent visits in the ED and the course of hospitalization.

**Results:**

Overall, 928 patients were included to the study and 101 (10.88%) patients were admitted to the ED more than once during three-month period. Patients visiting ED repeatedly were older (*p* = 0.03) and more likely to be hospitalized (*p* < 0.01) compared to single-visit patients. Patients during their subsequent visits spent more time in the ED (*p* = 0.01), had greater chance to repeat their appointment (*p* = 0.04), be admitted to the hospital (p < 0.01) and were more likely diagnosed with cholelithiasis (*p* = 0.03) compared to patients on their initial visit. If admitted to the surgical department they were also more often qualified for surgical procedure than patients on their first visit (p < 0.01). In a group of patients admitted to the surgical department there were no significant differences in rates of conversion, postoperative complications and mortality between subgroups.

**Conclusions:**

Readmissions among patients presenting with abdominal pain are a common phenomenon with prevalence of 10.88%. They are most commonly associated with cholelithiasis and occur more frequently among older patients, which suggests, that elderly require more attention during ED managements.

## Background

Emergency Department (ED) is an essential component of healthcare system. Number of patients treated at ED has been rising constantly [1]. It is necessary to maintain the highest quality of care despite growing number of patients.

Management of patients presenting with pain or tenderness in the abdominal area at the ED is conducted by various physicians including general surgeons. It is often challenging and it may be linked to increased rate of readmissions [2, 3].

Misdiagnosis, delayed treatment and inappropriate discharge advice was reported to occur in 50% of readmission cases [4]. Incidence of readmission to ED in a short period of time may signify that the previous evaluation of patient’s health status was inadequate [5]. Thus, frequent readmissions at EDs may be used as an indicator of low quality of care [4, 6]. The readmitted patients may be associated with increased complications and mortality rates compared to a single-visit patients [7, 8]. Moreover, often revisits generate higher costs than single-visit patients and contribute to overcrowding the ED [9].

Assessment of incidence and factors associated with readmissions of those patients may result in higher level of practitioners’ awareness and improvement of healthcare at the EDs.

## Methods

### Aim of the study

Our purpose was to assess the incidence of readmissions among patients treated in the ED due to abdominal pain. We also aimed to investigate the influence of ED readmission on their further course of treatment.

### Setting

The study was conducted at the ED of an academic center (tertiary referral level), which admits adult patients with illnesses or injuries requiring immediate medical attention. Patients are constantly supervised by at least 4 physicians working on call. Health benefits provided in this unit are preceded by medical segregation – TRIAGE system (confirmation or exclusion of an emergency health disorder and segregation of admitted patients based on the severity of their condition). TRIAGE is followed by full diagnostics and a necessary wide range of possible specialist consultations. The ED has access to a modern diagnostic imaging, equipment allowing for measurement of critical parameters and to a diagnostic laboratory with possibility of performing a full panel of tests. Treatment in the ED is conducted to the extent necessary for stabilizing the patient’s condition. Patients admitted to the ED may be transferred to nearly 40 clinical departments of an academic center, with whom the ED is constantly cooperating.

### Study design

We conducted a prospective observational study, which included patients admitted to the ED in one academic, teaching hospital presenting with non-traumatic abdominal pain in a three-month period (from January to March 2019). Inclusion criteria included presence of abdominal pain or abdominal tenderness during initial physical examination, age of 18 years old or higher, admission to the ED. Patients were selected by an ED physician who obtained necessary data with the use of computer software. The data was extracted at the end of the study period for further analysis. A 30-day follow-up period was additionally analyzed in order to assess the frequency of readmissions with highest possible precision. Study is designed and described regarding all STROBE checklist points for observational studies [10].

Patients were divided into two groups: patients who presented to the ED once and patients who presented more than once within a period of 30 days. Analyzed factors included age, sex, arrival and discharge time, length of stay in the ED, time since previous visit, additional tests taken in the ED (chest or abdomen radiograph, computed tomography (CT) scan, gastroscopy), ED discharge diagnosis, transfer to another department and in addition for those admitted to surgical ward: length of hospitalization, need and type of surgery, surgical complications rate, conversion rate, mortality and final diagnosis at the end of hospitalization. In analysis of subsequent visits to the ED we defined a readmission as a return of patient treated previously because of abdominal pain, reported again with the same or intensified symptoms within 30 days since previous discharge. Patients returning with other medical problem, not related to abdominal pain were excluded from group of patients with numerous visits. Additional tests were identified as diagnostic procedures not included in the standard protocol of care used in our ED including CT scan and gastroscopy.

### Analysis of endpoints

The primary endpoint was to determine the incidence of 30-day readmissions to the ED and compare patients admitted to the ED once and patients readmitted to the ED in terms of:

- age
- sex
- rate of hospital admissions
- rate of surgical ward admissions
- rate of non-surgical ward admissions

The secondary endpoint was to assess the differences between initial visit to the ED and every subsequent visit including:

- total time spent in the ED
- incidence of a subsequent visit
- number of performed additional tests
- proportion of patients admitted to the surgical ward
- proportion of patients qualified for surgery
- indications for surgical treatment
- conversions during surgery (from laparoscopy to laparotomy)
- postoperative complications
- mortality rate
- length of stay (LOS)

### Statistical analysis

Statistical data were calculated using StatSoft STATISTICA version 13. Shapiro-Wilk test was used for evaluating the normality of data distribution. Results were presented as a mean with standard deviation (SD) or median and interquartile range (IQR) for non-normally distributed values. To compare non-normally distributed data a non-parametric Mann-Whitney U test was used. During testing categorical variables, the Chi-square test of independence was applied. Results were considered statistically significant when *p* value was found to be < 0.05.

### Ethics approval and consent to participate

All procedures followed the ethical standards of the responsible committee on human experimentation (institutional and national) and 2013 Fortaleza revision of 1975 Declaration of Helsinki. Written informed consent was obtained from all participants.

## Results

### Material

**Fig. 1:**
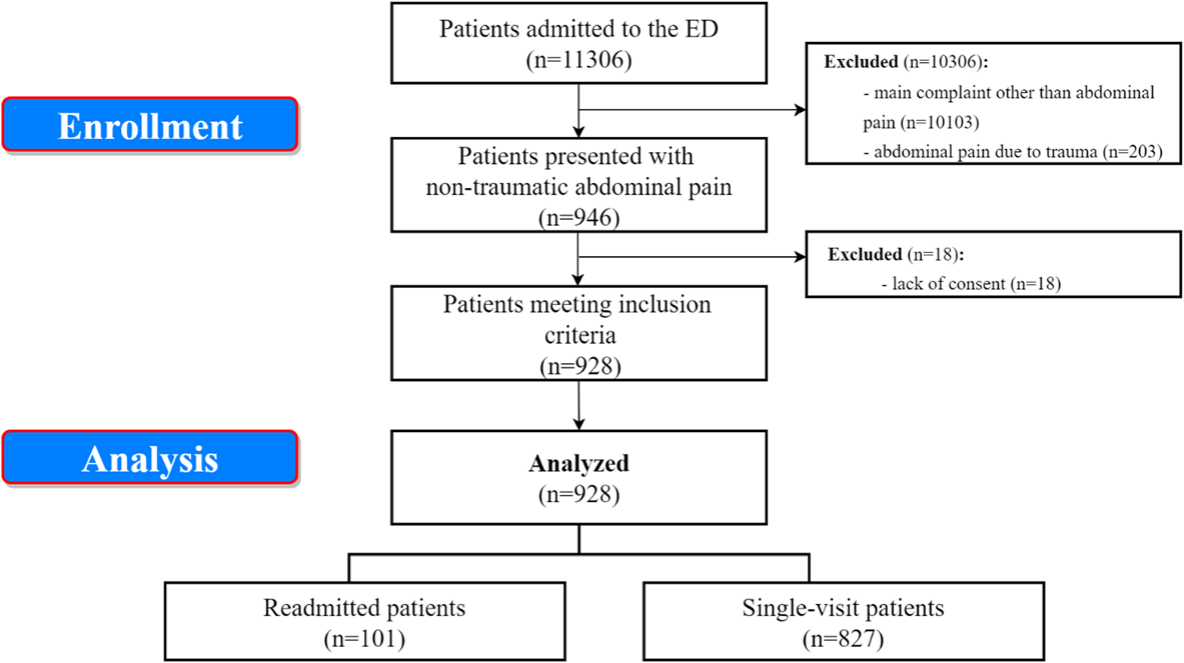
Study flowchart

In the 3-month study period, 11,306 patients were treated in the ED of our academic center. Among those, 928 patients met the study inclusion criteria (Fig. 1). Median age of the study group was 42 years (IQR: 26–62). It included 571 (61.53%) women and 357 (38.47%) men. Overall, 698 (75.22%) patients admitted to the ED were discharged home, 97 (10.45%) patients were hospitalized in a surgical ward and 133 (14.33%) were hospitalized in a non-surgical ward. The most frequent ED discharge diagnosis in whole group was “other or unidentified abdominal pain” in 52.59% of cases. In group admitted to surgical ward patients most often reported problems associated with biliary tract (28,87%), acute appendicitis (21,65%), obstruction (13,41%) and gastrointestinal hemorrhage (11,34%). Diagnosis classified as “other” (36,09%), unidentified abdominal pain (30,83%) and problems with biliary tract (16,54%) dominated among patients relocated to non-invasive treatment wards. Table 1 presents ED discharge diagnosis for all presented groups. (Table 1.)

**Table 1:** Study group characteristics

VARIABLES	ALL PATIENTS WITH ABDOMINAL PAIN	PATIENTS DISCHARGED HOME AFTER 1ST VISIT	PATIENTS REFERRED TO HOSPITAL	
			SURGICAL WARD	NON-INVASIVE TREATMENT WARD
**n** (% of all)	928 (100.00)	698 (75.22)	97 (10.45)	133 (14.33)
**age** - median (IQR)	42 (26–62)	38 (25–60)	55 (34–70)	58 (36–75)
**females** - n (% of group)	571 (61.53)	434 (62.18)	57 (58.76)	80 (60.15)
**males** - n (% of group)	357 (38.47)	264 (37.82)	40 (41.24)	53 (39.85)
**ED discharge diagnosis** - n (% of group)				
unidentified abdominal pain	488 (52.59)	441 (63.18)	6 (6.19)	41 (30.83)
renal and urinary disease	106 (11.42)	103 (14.76)	0 (0.00)	3 (2.26)
biliary tract related disease	85 (9.16)	36 (5.16)	27 (27.84)	22 (16.54)
GI tract bleeding	24 (2.59)	6 (0.86)	11 (11.34)	7 (5.26)
acute appendicitis	22 (2.37)	0	22 (22.68)	0 (0.00)
acute gastroenteritis	20 (2.16)	19 (2.72)	0	1 (0.75)
acute intestinal obstruction	14 (1.51)	0	14 (14.43)	0
peptic ulcer disease	9 (0.97)	3 (0.43)	6 (6.19)	0
gynaecological disease	6 (0.65)	3 (0.43)	0	3 (2.26)
neoplasm	6 (0.65)	4 (0.57)	0	2 (1.50)
other	148 (15.95)	83 (11.89)	11 (11.34)	54 (40.60)

IQR – interquartile range; ED – Emergency Department; GI – gastrointenstinal

### Primary endpoints

**Fig. 2:**
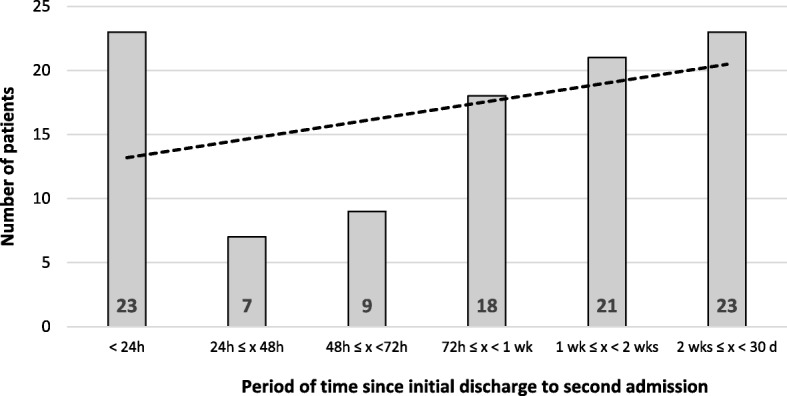
Distribution of patients depending of the period of time between initial discharge to the second admission to the ED (h – hour, d – day, wk. – week)

Overall, a group of 827 (89.12%) patients presented to the ED only once and 101 (10.88%) patients presented to the ED more than one time. A group of 23 (2.48%) patients were admitted for the second time during 24 h since initial discharge, 30 (3.23%) patients during 48 h, 39 (4.20%) patients during 72 h, 57 (6.14%) patients in one week, 78 (8.41%) patients in two weeks and 101 (10.88%) in 30 days. Figure 2. shows distribution of patients depending on the time since initial discharge to the second admission to the ED. In comparison with a single-visit group, multiple-visit patients were significantly older (*p* = 0.03). The rates of males and females in both groups was comparable (*p* = 0.64). Patients admitted to the ED only once were less likely to be hospitalized than those who were admitted multiple times (27.81% vs 44.55%, *p* < 0.01). Among patients admitted to the ED once, 84 (10.45%) were admitted to surgical ward and 126 (14.33%) were admitted to the non-surgical ward. Remaining 617 (75.22%) of patients were discharged from the hospital. In the group of patients admitted to the ED multiple times 36 (35.65%) of them were not admitted to hospital, 40 (39.60%) were admitted to surgical ward and 25 (24.75%) were admitted to non-surgical ward. (Table 2).

**Table 2:** Comparison of single- and multiple-admission patients (primary endpoints)

	Admissions to the ED:				p	OR	95% CI
	Single admission patients		Multiple admission patients				
	n	%	n	%			
**Number of patients**	827	89.12	101	10.88			
**Age** - years (IQR)	42	(26–62)	50	(32–65)	**0.0336**		
**Sex**							
Females	511	61.79	60	59.41	0.6430		
Males	316	38.21	41	40.59			
**Number of visits**							
1	827	100	–	–			
2	–	–	85	84.16			
3	–	–	12	11.88			
4	–	–	3	2,97			
5	–	–	1	0.99			
**Patients admitted to hospital**	210	27.81	65	64.35	**< 0.001**	5.31	3.43–8.21
**Patients admitted to surgical ward**	84	10.45	40	39.60	**0.0027**	2.40	1.36–4.25
**Patients admitted to** **non-invasive treatment ward**	126	14.33	25	24.75	**0.0156**	1.83	1.12–2.99

ED – Emergency Department; IQR – interquartile range

### Secondary endpoints

Patients during their subsequent visits spent more time in the ED than during their first visit [4 h (IQR: 3–6) vs. 4.68 h (IQR: 3–7, *p* = 0.01]. They also had greater chance to repeat their appointment [OR = 1.70 (95%CI: 1.02–2.85), *p* = 0.04] and greater chance of hospital admission [OR = 1.82 (95%CI: 1.22–2.71, *p* < 0.01)] compared to patients on their initial visit. Analysis revealed that patients admitted to surgical ward after their subsequent visit in the ED were more often qualified for surgical procedure than patients after their index visit [OR = 2.46 (95%CI: 1.40–4.32), *p* < 0.01]. In group of patients admitted to a surgical department, those after a single ED admission were most like to be qualified for surgery due to appendicitis (32.79%) followed by cholelithiasis/gallstones (18.03%). Patients admitted to surgical department after a multiple ED admissions were most likely to be qualified for invasive procedure due to cholelithiasis (44.44%) followed by appendicitis (16.67%). They were also characterized by longer LOS compared to patients admitted after index ED visit [median time (days): 3 (2–5) vs. 4 (3–6), *p* = 0.04]. There were no significant differences in rates of conversion (*p* = 0.78), postoperative complications (*p* = 0.82) and mortality (*p* = 0.96) between those two groups. (Table 3.).

**Table 3:** Comparison of initial visit and readmission to the ED (secondary endpoints)

	Initial visit		Readmission		p	OR	95% CI
No of visits - n (% of all visits)	928	88.38%	122	11.62%			
**Total time spent on the ED** - hours (IQR)	4	(2.75–6.04)	4.68	(3.48–6.98)	**0.0054**		
**Not the last visit** - n repeated visits (% of visits)	101	10.88%	21	17.21%	**0.0422**	1.7	1.02–2.85
**Number of additional imaging tests** - n (% of visits)							
0	889	95.80%	115	94.26%			
1	39	4.20%	6	4.92%	0.6897		
2	0	0%	1	0.82%			
**Admission to hospital ward** –n (% of visits)	230	24.78%	47	38.52%	**0.0014**	1.90	1.28–2.82
**Admission to surgical ward** - n (% of visits)	97	10.45%	29	23.77%	**< 0.001**	2.67	1.67–4.26
**Qualification for invasive surgical treatment** - n (% of group)	61	6.57%	18	16.83%	**0.0017**	2.46	1.40–4.32
**Indication**							
**Cholelithiasis/gallstones**	11	18.03%	8	44.44%	**0.0259**	3.64	1.17–11.32
**Appendicitis**	20	32.79%	3	16.67%	0.1859	0.41	0.11–1.58
**Hernia**	8	13.11%	0	0.00%	0.2314	0.17	0.01–3.09
**Acute pancreatitis**	7	11.48%	1	5.56%	0.5740	0.53	0.06–4.68
**Obstruction**	4	6.56%	1	5.56%	0.8782	0.83	0.09–8.01
**Neoplasm**	3	4.92%	1	5.56%	0.9137	1.13	0.11–11.65
**Peptic ulcer**	3	4.92%	0	0.00%	0.6047	0.45	0.02–9.15
**Other**	5	8.20%	4	22.22%	0.1132	3.20	0.76–13.50
**Conversion** - n (% of group)	2	3.28%	0	0.00%	0.7789	0.64	0.03–14.01
**Postoperative complications** - n (% of group)	8	13.11%	2	11.10%	0.8225	0.82	0.16–4.30
**Death** - n (% of group)	1	1.03%	0	0.00%	0.9584	1.09	0.04–27.91
**Length of hospital stay** - days (IQR)	3	(2–5)	4	(3–6)	**0.0443**		

ED – Emergency Department; IQR – interquartile range

## Discussion

Abdominal pain seems to be one of the most important medical problems associated with high risk of readmissions to the ED [11]. In our study approximately one of every ten patients presenting with abdominal pain was readmitted to the ED during 30 days after initial visit. Depending on a study design ED readmission rate varied from 0.39% to even 49.3%, which resulted mainly from different readmission time frames (range 48 h to 365 days) and characteristics of the study group [11, 12]. We found two studies investigating readmissions after 30 days since initial visit: Patterson et al. estimated it as 12.4% among patients with abdominal pain and Friedman et al. assessed it as 12% among older adults [13, 14] In Meltzer’s study focused on patients with abdominal pain and 365-day readmission rate was 41% [12]. Our revisit rates of 2.48, 4.14, 6.20% in consecutive time frames of 48 h, 72 h and one week are consistent with previous findings [15].

Patients with more than one visit at the ED were significantly older than single-visit patients, which was also proved in previous publications [14, 16–20]. In the study by Hu et al. old age was identified as an independent risk factor, not deriving from higher incidence of comorbidities [17]. Gabayan et al. described predictors of readmission in adults such as older age, skilled nursing facility use, leaving the ED against medical advice and chronic conditions such as renal disease and heart failure [21]. In our study group majority of readmitted patients were subsequently admitted to a surgical ward. It is associated with most common diseases diagnosed among those patients, which were appendicitis and biliary tract diseases - ailments that are often misdiagnosed and recurring [22–24].

Previous studies present abdominal pain as a one of the most often symptoms observed in readmitted patients with prevalence ranging from 15.5 to 29.1%, as well as the most common complaint leading people to the EDs [4, 8, 16, 25, 26]. In our study the most common discharge diagnosis after readmission to the ED visit was cholelithiasis. Furthermore, cholelithiasis was observed significantly more often in patients during their subsequent admission to the ED. It may be explained by recurrent symptoms, which are often observed by patients who have recently experience biliary colic symptoms [27]. Williams et al. showed that failure to achieve a timely surgical follow-up in this group of patients may result in multiple ED readmissions and emergent gallstone-related hospitalizations [28]. Appendicitis, which was the second most common final diagnosis during subsequent visits was the reason for readmission of three patients. Delayed diagnosis was associated with significantly higher rate of hospital-admissions, more frequent need of invasive treatment and longer LOS. Postoperative complication rates were comparable in both groups.

Imaging techniques have been more frequently used in management of patients with abdominal pain, however have brought minor advantages in diagnostic specificity [26, 29, 30]. Medford-Davis et al. presented high incidence of diagnostic errors reaching 35% in high risk patients with abdominal pain, involving most commonly history taking, but also ordering insufficient tests and problems with follow-up of abnormal test results [31]. Nonetheless, Patterson et al. proved effectiveness of CT imaging in reduction of 30-day revisit rate of patients with non-traumatic abdominal pain [14]. The number of performed imaging examinations did not differ between initial and subsequent admissions and did not influence the chance of readmission.

Subsequent admissions lasted significantly longer compared to the initial ones, which may contribute to increased overcrowding of EDs [32]. Cheng et al. suggested that initial visits may be shorter because ED doctors want to prevent overcrowding and patients do not wish to stay in observational room after achieving the relief of symptoms after initial treatment [33]. Therefore, the premature discharge may be related to inadequate treatment and be partially responsible for readmissions.

Our study is associated with several limitations. It is a prospective observational study based on ED and surgical department medical records. The study was carried out in only one center and the results may not be generalized to other setting, as the impact of demographic factors cannot be assessed. Furthermore, some of the patients may have been admitted to another ED after being admitted in our department. However, we believe that the rate of crossover cases is low and randomly distributed among groups. Further research needs to be conducted on larger group of patients and preferably include multiple centers located in close proximity to prevent the crossover bias.

## Conclusion

Readmissions among patients presenting with abdominal pain are a common phenomenon with prevalence of 10.88%. Readmitted patients spend more time in the ED and are more often admitted to the hospital. They are also more likely to be qualified for invasive surgical treatment with comparable outcomes as a single-visit patients, except for a longer LOS. Readmissions are most commonly associated with cholelithiasis and occur more often among older patients, which suggests that elderly require more attention during ED hospitalization.

## Data Availability

Data are available from the authors upon reasonable request.

## Abbreviations

ED: Emergency Department
LOS: Length of stay
CT: Computed tomography
